# Hybrid Hypercube Optimization Search Algorithm and Multilayer Perceptron Neural Network for Medical Data Classification

**DOI:** 10.1155/2022/1612468

**Published:** 2022-03-25

**Authors:** Mustafa Tunay, Elnaz Pashaei, Elham Pashaei

**Affiliations:** ^1^Department of Computer Engineering, Istanbul Gelisim University, Istanbul, Turkey; ^2^Department of Software Engineering, Istanbul Aydin University, Istanbul, Turkey

## Abstract

The hypercube optimization search (HOS) approach is a new efficient and robust metaheuristic algorithm that simulates the dove's movement in quest of new food sites in nature, utilizing hypercubes to depict the search zones. In medical informatics, the classification of medical data is one of the most challenging tasks because of the uncertainty and nature of healthcare data. This paper proposes the use of the HOS algorithm for training multilayer perceptrons (MLP), one of the most extensively used neural networks (NNs), to enhance its efficacy as a decision support tool for medical data classification. The proposed HOS-MLP model is tested on four significant medical datasets: orthopedic patients, diabetes, coronary heart disease, and breast cancer, to assess HOS's success in training MLP. For verification, the results are compared with eleven different classifiers and eight well-regarded MLP trainer metaheuristic algorithms: particle swarm optimization (PSO), biogeography-based optimizer (BBO), the firefly algorithm (FFA), artificial bee colony (ABC), genetic algorithm (GA), bat algorithm (BAT), monarch butterfly optimizer (MBO), and the flower pollination algorithm (FPA). The experimental results demonstrate that the MLP trained by HOS outperforms the other comparative models regarding mean square error (MSE), classification accuracy, and convergence rate. The findings also reveal that the HOS help the MLP to produce more accurate results than other classification algorithms for the prediction of diseases.

## 1. Introduction

Medical data classification is a growing field of research that provides pathologists with vital knowledge for diagnosing and treating diseases. Multilayer perceptron (MLP) neural network is one of the most powerful classification algorithms that has been successfully and widely employed for many medical problems [1–4]. The key point in using MLP is to determine the best values for the parameters of the neural network. The most commonly used gradient-based learning method of MLP is the backpropagation (BP) algorithm. However, this learning method has some flaws, including sluggish convergence, high reliance on the initial solutions, and a proclivity for getting stuck in local optima. Optimization algorithms (OAs) are offered as viable alternatives to gradient-based MLP training approaches in this regard. Several works have been published in the literature, including group search optimizer (GSO) [5], symbiotic organisms search (SOS) [6] algorithm, lightning search algorithm (LSA) [7], ant lion optimizer (ALO) [8], Krill herd algorithm (KHA) [9], grasshopper optimization algorithm (GOA) [10, 11], artificial bee colony (ABC) [12], social spider optimization algorithm (SSO) [13], hybrid of ABC and dragonfly algorithm (DA) [14], artificial ant colony optimization (ACO) [15], particle swarm optimization (PSO) [16], cuckoo search (CS) [17, 18], moth-flame optimization (MFO) [19, 20], whale optimization algorithm (WOA) [21], gray wolf optimizer (GWO) [22, 23], black hole algorithm (BHA) [24], invasive weed optimization [25], multiverse optimizer algorithm (MOA) [26, 27], bat algorithm [28], and salp swarm algorithm (SSA) [29].

Although various OAs have already been investigated for training MLP neural networks, because of the duality of Aos' exploration and exploitation capabilities, there is still room for new designs and upgrades to current ones [30]. Also, in training MLP, the issue of slow convergence rate and trapping in local optima remains partially unsolved.

The purpose of this study is to introduce a new optimization technique, called the hypercube optimization search (HOS) algorithm, for training MLP to present an improved classification approach for medical data by optimizing the MLP's weights and biases parameters. The HOS is recommended for training MLP to overcome the aforementioned challenges due to its outstanding performance in escaping local optima and fast convergence speed [31, 32]. Also, HOS have fewer parameters and is easy to use, simple in principle, and adaptable when compared to other swarm-based OAs.

This paper's contributions can be summed up as follows:

- To propose a new stochastic learning approach for training MLPs, in order to boost the MLP's performance in the classification of health data.
- To evaluate HOS-MLP's performance on four important medical datasets: diabetes, breast cancer, coronary heart disease, and orthopedic patients, and compare its performance against eleven different classifiers and eight well-known OA-based MLP trainer techniques.
- To achieve better outcomes than previous studies, using suggested HOS-MLP in terms of mean square error (MSE), classification accuracy, and convergence rate.

This paper is structured as follows: Section 2 presents the MLP. The HOS algorithm is explained in Section 3, whereas the proposed HOS-MLP approach is introduced in Section 4.  Section 5 shows the experimental results and discussion. Finally, section 6 gives a conclusion as well as recommendations for further work.

## 2. Multilayer Perceptron Neural Network

**Figure 1:**
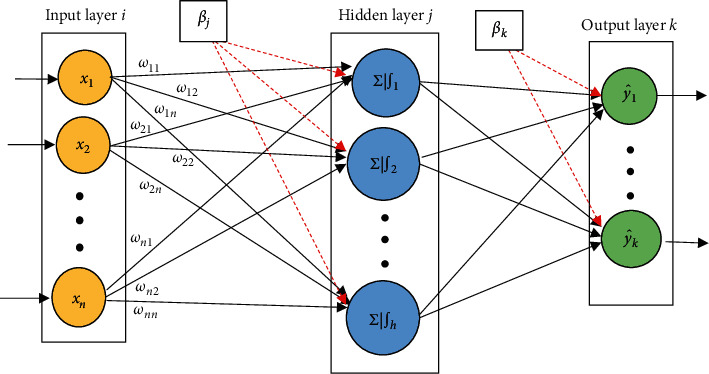
Structure of single hidden layer MLP.

The feedforward neural network (FNN) is one of the most prevalent forms of artificial neural network (ANN) and MLP is a well-known type of FNN that is widely used in solving realistic classification problems [10]. An MLP is made up of three groups of layers: input (*i*), hidden layer (*j*), and output (*k*). Each layer consists of a specific number of neurons, and each neuron has full-weighted connections with the adjacent layer neurons. A single hidden layer MLP network was used in this paper, which was demonstrated in Figure 1.

Each neuron can carry out two functions in the MLP: weighted summation and activation. The weighted sum is calculated using equation (1) for each hidden neuron *j* as follow:

(1) $$\begin{array}{c} s_{j}=\overset{n}{\underset{i=1}{\sum }}w_{ij}x_{i}+\beta_{j}, \end{array}$$

where *w*_*ij*_ describes the connection weight, *β*_*j*_ is the biased term, *x*_*i*_ denotes the input *i*, and *n* shows the total number of inputs. In the second step, using the outcome of equation (1), an activation function is utilized to calculate the neurons' output. The function is illustrated as follows:

(2) $$\begin{array}{c} f\left(s_{j}\right)=\frac{1}{1+e^{-s_{j}}}. \end{array}$$

The most commonly used sigmoid activation function was selected in the MLP [22, 26]. Utilizing the results of the hidden neurons, the final productions of the output neurons are computed as follows:

(3) $$\begin{array}{c} y_{k}=f\left(o_{k}\right)=\frac{1}{1+e^{-s_{k}}},\\ s_{k}=\overset{h}{\underset{j=1}{\sum }}w_{jk}f\left(s_{j}\right)+\beta_{k}. \end{array}$$

MLP's performance depends highly on weights and biases, and training MLP aims to find optimal weights and biases. MLP training is a challenging task that leads to a high-performance MLP [21]. The HOS algorithm is used as a training method for MLPs in the following sections.

## 3. Hypercube Optimization Search Algorithm

The HOS algorithm, inspired by a dove's behavior in exploring new food zones, was proposed by Abiyev and Tunay for solving high-dimensional numerical problems [31]. The HOS algorithm is based on a randomly distributed set of points inside an m-dimensional hypercube (HC). HOS exhibit fast population convergence by shrinking the area of the HC at each iteration. The HOS algorithm consists of three stages: (A) the initialization process, (B) the displacement-shrink process, and (C) the searching areas process. These stages can be described in detail as follows.

### 3.1. Stage A: Initialization Process

The HOS algorithm begins with the initialization process, in which randomly generated points within a given HC form the candidate solutions matrix. Several starting conditions in the initialization phase should be computed, including (1) lower-upper boundaries (*lb*, *ub*), (2) size ( *r*_*di* *m*_), (3) central value (*x*_*c*_), and (4) dimension of the HC (*m*).

(4) $$\begin{array}{c} m\;=\text{ length }\left(x_{c}\right),\\ x_{c}=\frac{\left(ub+\;lb\right)}{2}\text{,}\\ d=lb\;-\;ub\;,r_{di\;m}=\frac{d}{2}\text{,}\\ \;lb=\text{ min }\left(X\text{ bounds}\right),\\ ub=\text{ max }\left(X\text{ bounds}\right).\; \end{array}$$

At the starting stage, the first HC is created by assigning random values to *r*_*di* *m*_ and *x*_*c*_. The uniformly distributed *N* points *x*_*i*_=(*x*_*i*1_, *x*_*i*2_,…, *x*_*im*_) are then randomly produced inside the HC. These points could also be represented in matrix form *X* with the size of (*N* × *m*). The upper and lower boundaries of the first HC are then calculated using the *X* matrix. The *r*_*di* *m*_ and *x*_*c*_ of the next HC are determined using those boundaries. The *X* matrix is also utilized for evaluation, in which the best value of the fitness function *F*_*best*_ and corresponding *x*_best_ point is determined within the population at *i* th iteration. Using local search, the *x*_best_ point is improved as follows:

(5) $$\begin{array}{c} x_{\text{best}}^{\text{new}}=x_{\text{best}}+\rho \Delta F, \end{array}$$

where *F* is the fitness function, and 0 ≤ *ρ* ≤  1.

### 3.2. Stage B: Displacement-Shrink Process

The displacement-shrink phase aims to determine the center of the next hypercube (new hypercube) *x*_*c*_*new*__ and evaluate the fitness function. The center of the next hypercube is obtained using the average of the sum of the previous hypercube's center and the present best point (*x*_best_) as follows:

(6) $$\begin{array}{c} x_{c_{\text{new}}}=\frac{x_{\text{best}}^{\text{new}}\;+\;x_{c}}{2}. \end{array}$$

In this process, each iteration generates fresh data points, and the fitness function is evaluated. The hypercube size has been modified based on the evaluation results. This process is used as a conservative measure to reduce excessive variability in the search space. As a consequence, the size of HC is decreased and the search space is reduced, which is called “shrinking.” The density of the search points (population) increases as the hypercube size decreases. The movement of the best value is governed by contraction. For smaller movements, the contraction is stronger. This ensures rapid convergence while also preventing the algorithm from becoming trapped at an undesirable (local) minimum.

The algorithm will cycle through a sequence of points starting from the current position to estimate the maximum distance. The value of the *F*_*best*_ is first compared with the *F*_mean_=*F*((*x*_best_+*x*_last−center_) /2). If *F*_*mean*_ value is less than *F*_*best*_ in the given iteration, *x* displacements (or *x* movements) is computed and normalized twice at each iteration using the following formulas:

(7) $$\begin{array}{c} \text{Normalized }x\left(\text{the previous }x\text{ for minimum}\right)x_{n}=\frac{x-x_{c}}{d}, \end{array}$$

(8) $$\begin{array}{c} \text{Normalized }x_{min}\;\left(\text{current }x\text{ for minimum}\right)x_{n}=\frac{x_{min}-x_{c}}{d}, \end{array}$$

(9) $$\begin{array}{c} \text{Normalize distance }d_{n}=\frac{\text{sum}{\left({\left(x_{n}-x_{\min n}\right)}^{2}\right)}^{0.5}}{d}, \end{array}$$

(10) $$\begin{array}{c} \text{Re}-\text{normalize distance }d_{nn}=\frac{d_{n}}{\sqrt{m}}. \end{array}$$

To convert the displacement into unity-sided points, each element of *x* is first divided by the associated beginning interval (equations (7) and (8)), and then this number is again normalized by dividing it to the diagonal of the points, i.e., $\sqrt{m}$ (equations (9) and (10)). If *F*_*mean*_ value in the specified iteration is greater than *F*_*best*_, *x* displacements will not occur and *d*_*nn*_ will be assigned to 1. The searching areas process is carried out in the next step if the conditions are not met.

### 3.3. Stage C: Searching Areas Process

The phase of the search area generates a new HC by initializing new values to *r*_*di* *m*_ and *x*_*c*_ according to the value of *d*_*nn*_. If the 0 ≤ *d*_*nn*_ ≤ 1 condition is satisfied, the factor of convergence *S* is calculated and values of the *r*_*di* *m*_ and *x*_*c*_ are updated accordingly using the following formulas:

(11) $$\begin{array}{c} x_{c}=x_{\text{best}},\\ \;r_{\text{dim}}=\;r_{\text{dim}}*S,\\ S=1-0.2e^{-3d_{nn}}. \end{array}$$

**Figure 2:**
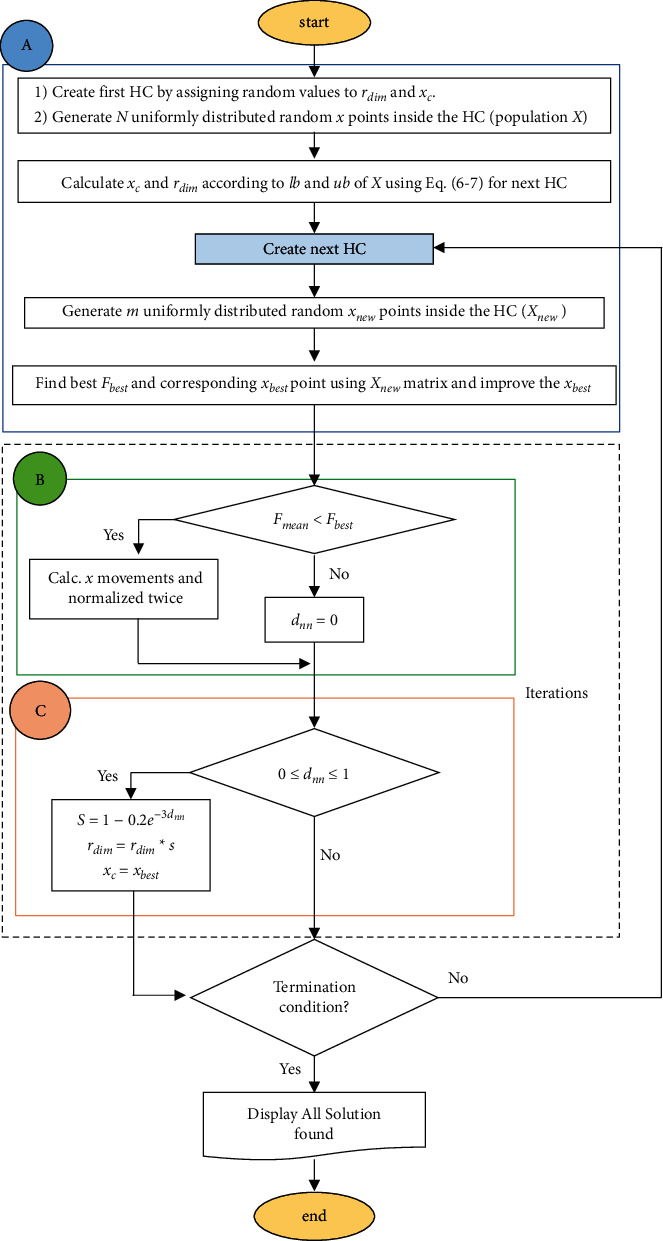
The flowchart of the HOS algorithm.

The size of the HC is reduced by multiplying  *r*_*di* *m*_ with *S* factor. If 0 ≤ *d*_*nn*_ ≤ 1 condition is met, the size of HC remains unchanged. HOS ensure the quick arrival of candidate solutions to a global minimum by reducing the area of the hypercube after each iteration. The entire procedure is repeated till particular termination criteria are met. The HOS algorithm is depicted in Figure 2. More details are provided in [31, 32].

## 4. HOS for MLP Training

The suggested HOS-MLP method, in which the HOS algorithm is utilized for training the MLP, is explained in detail in this section. When the method is designed, two important aspects are considered: (1) the representation of candidate solutions in HOS for training MLP, and (2) the definition of a fitness function for solution assessment.

The matrix encoding approach is utilized in HOS-MLP to represent candidate solutions. For MLP's weight and bias parameters, each solution provides a set of values. A solution can be represented as follows:

(12) $$\begin{array}{c} W_{1}=\left[\begin{array}{ccc} w_{1,1} & \cdots & w_{n,1}\\ \vdots & \ddots & \vdots \\ w_{1,h} & \cdots & w_{n,h} \end{array}\right],\\ \beta_{1}=\left[\begin{array}{c} \beta_{1}\\ \vdots \\ \beta_{h} \end{array}\right],\\ W_{2}^{\text{'}}=\left[\begin{array}{ccc} w_{1,1} & \cdots & w_{h,1}\\ \vdots & \ddots & \vdots \\ w_{1,k} & \cdots & w_{h,k} \end{array}\right],\\ \;\beta_{2}=\left[\begin{array}{c} \beta_{1}\\ \vdots \\ \beta_{k} \end{array}\right],\\ {\text{solution}}_{i}=\left[W_{1},\;\beta_{1},W_{2}^{\text{'}},\beta_{2}\right]. \end{array}$$

where *W*_1_ indicates the weight matrix of linkages between hidden neurons and input and *W*_2_′ demonstrates the transpose of the weights matrix of the linkages between the hidden neurons and output. For hidden and output neurons, the *β*_1_, and *β*_2_ represent bias values, respectively.

It is worth mentioning that the number of neurons in the input and output layers is specified by the dataset's total number of features and labels, while the Kolmogorov theorem is utilized to determine the number of neurons within the hidden layer (H) using the following equation:

(13) $$\begin{array}{c} \text{H}=2*\text{Input}+1. \end{array}$$

The MSE is utilized as the objective function for measuring the fitness value of candidate solutions in the proposed HOS-MLP approach as follows:

(14) $$\begin{array}{c} \text{MSE}=\frac{1}{n}\;\overset{n}{\underset{i=1}{\sum }}{\left(y-\hat{y}\right)}^{2}, \end{array}$$

where *y* and $\hat{y}$ symbolize the actual and predicted class label, and *n* is the number of samples in training data. The HOS based MLP training approach is carried out in the following stages:

1. Initialization: within an HC, the initial solutions (points) are generated randomly. Each solution represents the possible values for the parameters of MLP.
2. Fitness evaluation: the solutions are assigned to MLPs, and the goodness of each MLP is then evaluated using MSE. The objective is to find the best MLP with the least MSE based on the dataset's training samples.
3. Creating new HC and update solutions.
4. Steps 2 and 3 are repeated until the full number of iterations is completed. Eventually, the solution with the least MSE is reported as the optimal solution. It is then allocated to MLP and the performance of the trained MLP is assessed on the test dataset.

**Figure 3:**
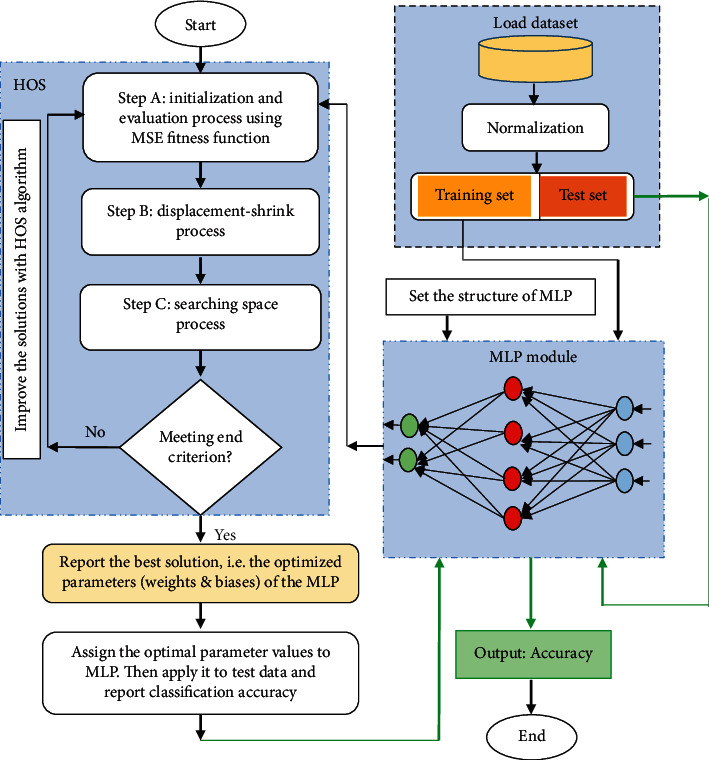
The flowchart of suggested HOS-MLP for medical data classification.

Figure 3 illustrates the suggested HOS-MLP framework.

## 5. Results and Discussions

In this section, the proposed HOS-MLP model is examined on four medical datasets: orthopedic patients (vertebral column) [33], diabetes [34], coronary heart disease (Saheart) [35], and Wisconsin breast cancer [36]. The characteristics of the medical datasets are summarized in Table 1.

All medical datasets are split into two parts: 66.66% of the data is used for the training set, and the remaining (33.33%) is used for the test set. In this partitioning, stratified sampling is used to retain the initial data distribution in the training and testing. The algorithms have been run 20 different times to produce statistically valid results. The Min-max scaling method was utilized to standardize all feature values within the range [0, 1] using the following equation:

(15) $$\begin{array}{c} x^{\prime }=\frac{x_{i}-\min}{\max-\min}. \end{array}$$

The suggested HOS-MLP is compared with eight well-known and recent Oas, including ABC [12], PSO [16], BAT [28], GA [37], BBO [38], firefly algorithm (FF/FA) [39], monarch butterfly optimization (MBO) [40], and flower pollination algorithm (FPA) [41]. For all OAs, the population size was set to 70, and the maximum number of iterations was set to 250 in all experiments. Two optional parameters in the HOS algorithm, *tolF*, and *tolX*, were set to 1e-09 and 1e-01, respectively. The parameters *tolF* and *tolX* represent relative tolerance for fitness function and vector *x* to stop the algorithm. The evaluation measures employed in this work are accuracy, MSE, box plot, and coverage rate. The rest of the parameters were set as suggested in [42].

### 5.1. Breast Cancer Dataset

**Figure 4:**
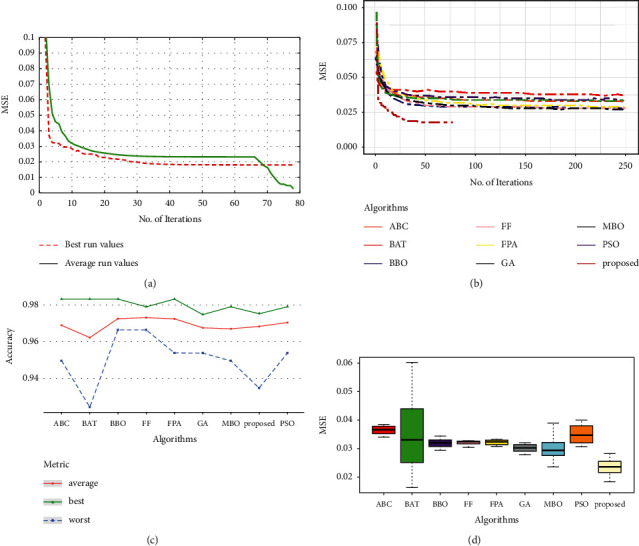
Performance analysis of the breast cancer dataset: (a) convergence curve of the proposed approach, (b) convergence curve for the compared methods, (c) accuracy for the compared methods, and (d) box plot for the compared methods.

Many binary classification problems use accuracy and MSE metrics to show the model's ability to split the two-class labels.  Table 2 summarizes the testing set results for the suggested HOS-MLP model compared to other OAs models from the literature. From Table 2, Figures 4(a) and 4(b), it can be noticed that the suggested method performs very better than other methods in terms of convergence rate. Although all algorithms achieved high ratios in terms of average accuracy, the suggested HOS-MLP shows reasonable and competitive results with the lowest MSE average (Figures 4(c) and 4(d)).

### 5.2. Diabetes Dataset

**Figure 5:**
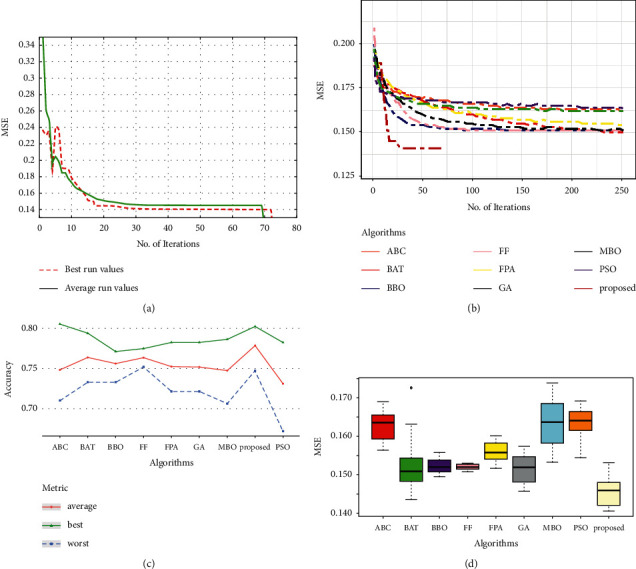
Performance analysis of the diabetes dataset: (a) convergence curve of the proposed approach, (b) convergence curve for the compared methods, (c) accuracy for the compared methods, and (d) box plot for the compared methods.

The diabetes dataset evaluation results are illustrated in Table 3 and Figure 5. When the convergence curves in Figure 5(a) and 5(b) are compared to the other algorithms, the suggested strategy has a very high convergence rate, while most methods, such as GOA and ABC, have stagnated after 98 iterations. The proposed approach displays the maximum ratios in terms of average and best accuracy (Table 3 and Figure 5(c)). The boxplot (Figure 5(d)) indicates that, while GOA has a more compact box, the proposed approach has the lowest error and acceptable stability.

### 5.3. Saheart Dataset

**Figure 6:**
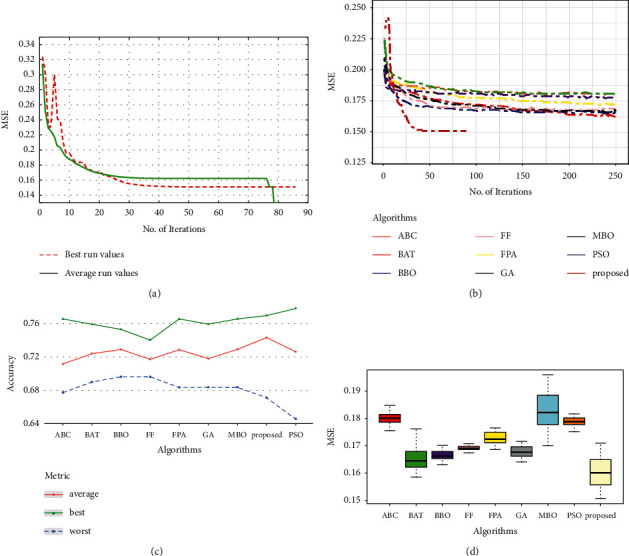
Performance analysis of the Saheart dataset: (a) convergence curve of the proposed approach, (b) convergence curve for the compared methods, (c) accuracy for the compared methods, and (d) box plot for the compared methods.

Comparing the HOS-ML model with other OAs models from Table 4, we obtained better accuracy and MSE. This observation proves that HOS-ML can accurately model classification tasks.  Figure 6 demonstrates the proposed HOS-ML model's accuracy, MSE, convergence speed, and stability. In terms of convergence speed, Figures 6(a) and 6(b) illustrate that, relative to the other algorithms, the proposed MLP-based trainer has a very fast convergence rate and the smallest MSE average (see Figure 6(d)). The suggested strategy produces an improved performance in contrast to other methods in terms of average accuracy (Figure 6(c)).

### 5.4. Vertebral Dataset

**Figure 7:**
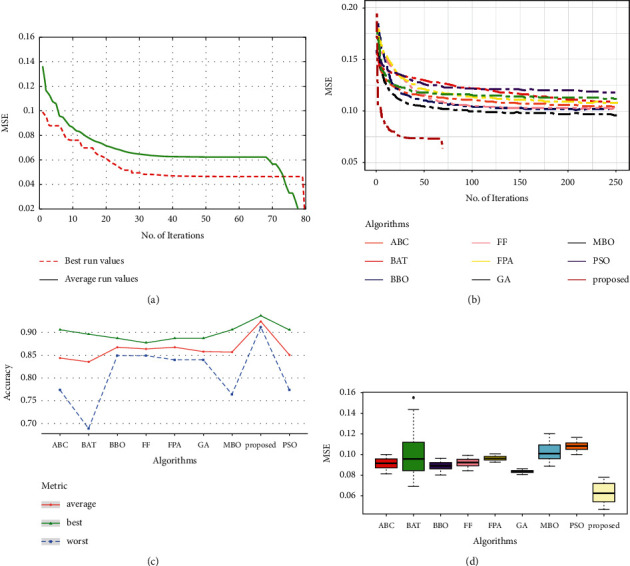
Performance analysis of the vertebral dataset: (a) convergence curve of the proposed approach, (b) convergence curve for the compared methods, (c) accuracy for the compared methods, and (d) box plot for the compared methods.

The results of the evaluations for the vertebral dataset is shown in Table 5 and Figure 7. For this dataset, the evaluation results of all MLP-trainers were very close and competitive, but our proposed approach showed very faster convergence as can be seen in Figures 7(a) and 7(b). The boxplot (Figure 7(d)) also confirms that the proposed approach has the smallest MSE. Moreover, our suggested algorithm has obtained outstanding performance in terms of worst, average, and best accuracy (Table 5 and Figure 7(c)).

**Figure 8:**
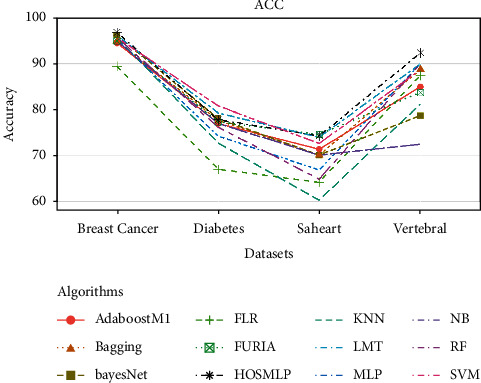
The classification performance of twelve classifiers on 4 medical datasets.

The average classification accuracy of eleven different classifiers on 4 medical datasets is shown in Table 6 and Figure 8. These classifiers are Naïve Bayes (NB), Bayes network learning (BayesNet), support vector machine (SVM) [43, 44], MLP using backpropagation (MLP), K nearest neighbor (KNN), AdaboostM1 [45], bagging, fuzzy lattice reasoning (FLR) classifier, random forest (RF) [46], fuzzy unordered rule induction algorithm (FURIA), and logistic model tree (LMT).

As shown in Table 6 and Figure 8, the proposed algorithm has the best performance among the eleven algorithms on 3 medical datasets. For the diabetes dataset, the proposed HOS-ML ranked 4^th^, after SVM, BayesNet, and LMT.

Overall, the experimental findings demonstrate that the MSE results of the proposed HOS-MLP are greatly better relative to other MLP-based optimization techniques for all medical datasets. The outstanding advantage of HOS is that it can achieve accurate results with a significantly higher convergence rate than other existing methods. However, some parameters in HOS should be adjusted, and some elements of HOS can be tweaked to increase the algorithm's classification accuracy in certain datasets.

## 6. Conclusion

This study introduced an improved classification approach, HOS-MLP, to increase the precision of medical diagnosis. The HOS algorithm was employed to adjust the MLP weights and bias values. The high-performance, simplicity, and fast convergence speed of the HOS algorithm were the inspiration behind the choice of HOS for training MLP. To evaluate the efficacy of the suggested HOS-MLP, its classification performance was assessed on four challenging real biomedical datasets: coronary heart disease, orthopedic patients, diabetes, and breast cancer. The performance of the model was compared with eleven different classifiers and eight well-known OA-based MLP-trainers such as ABC, GA, BAT, BBO, PSO, FF, FPA, and MBO. The experimental results of HOS on those biomedical classification problems are promising in terms of convergence rate compared to existing OAs. It managed to demonstrate better classification accuracy in most cases. We conclude that the HOS can train MLP well for classifying biomedical datasets since the HOS-trained MLP presents a higher convergence speed and better classification accuracy than current MLP training techniques and existing state-of-the-art classifiers.

In future work, HOS can be utilized to find the optimal structure of the MLP neural network, including the number of hidden layers and nodes. HOS can also be employed to train other forms of ANNs, such as the radial basis function (RBF). It may also be a valuable contribution to solving engineering classification problems using the proposed HOS-MLP.

## Figures and Tables

**Table 1:** Datasets description.

Dataset	#Features	#Class	#Training sample	#Testing sample	MLP structure (input-hidden-output)
Breast cancer	9	2	469	230	9-19-2
Diabetes	8	2	515	253	8-17-2
Saheart	9	2	310	152	9-19-2
Vertebral column	6	2	161	79	6-13-3

**Table 2:** The performance results of comparative methods on the breast cancer dataset in the terms of mean square error (MSE) and classification accuracy.

Comparative algorithms	MSE					Accuracy (%)	
	Best	Worst	Mean	Best		Worst	Mean
ABC-MLP	0.03400	0.03843	0.03639	98.319		94.958	96.891
BAT-MLP	0.01624	0.06023	0.03432	98.319		92.437	96.218
BBO-MLP	0.02938	0.03442	0.03195	98.319		96.639	97.255
FF-MLP	0.03043	0.03279	0.03209	97.899		96.639	97.311
FPA-MLP	0.03079	0.03320	0.03216	98.319		95.378	97.241
GA-MLP	0.02786	0.03206	0.03020	97.479		95.378	96.751
MBO-MLP	0.02351	0.03887	0.02974	97.899		94.958	96.695
PSO-MLP	0.03062	0.03999	0.03496	97.899		95.378	97.045
HOS-MLP	0.01833	0.02819	0.023464	97.521		93.4733	96.833

**Table 3:** The performance results of comparative methods on the diabetes dataset in the terms of mean square error (MSE) and classification accuracy.

Comparative algorithms	MSE				Accuracy (%)	
	Best	Worst	Mean	Best	Worst	Mean
ABC-MLP	0.15635	0.16900	0.16276	80.534	70.992	74.822
BAT-MLP	0.14357	0.17267	0.15299	79.389	73.282	76.374
BBO-MLP	0.14947	0.15574	0.15230	77.099	73.282	75.611
FF-MLP	0.15074	0.15300	0.15199	77.481	75.191	76.349
FPA-MLP	0.15161	0.16008	0.15582	78.244	72.137	75.242
GA-MLP	0.14571	0.15739	0.15156	78.244	72.137	75.165
MBO-MLP	0.15326	0.17385	0.16355	78.626	70.611	74.733
PSO-MLP	0.15438	0.16916	0.16372	78.244	67.176	73.104
HOS-MLP	0.14051	0.15314	0.14548	80.237	74.701	77.840

**Table 4:** The performance results of comparative methods on the Saheart dataset in the terms of mean square error (MSE) and classification accuracy.

Comparative algorithms	MSE					Accuracy (%)	
	Best	Worst	Mean	Best		Worst	Mean
ABC-MLP	0.17556	0.18474	0.18007	76.582		67.722	71.160
BAT-MLP	0.15843	0.17620	0.16510	75.949		68.987	72.405
BBO-MLP	0.16308	0.17000	0.16646	75.316		69.620	72.911
FF-MLP	0.167397	0.17072	0.16916	74.051		69.620	71.730
FPA-MLP	0.16852	0.17657	0.17273	76.582		68.354	72.869
GA-MLP	0.16391	0.17165	0.16776	75.949		68.354	71.814
MBO-MLP	0.17005	0.19585	0.18274	76.582		68.354	72.932
PSO-MLP	0.17505	0.18158	0.17867	77.848		64.557	72.658
HOS-MLP	0.15063	0.17096	0.16015	76.973		67.101	74.842

**Table 5:** The performance results of comparative methods on the vertebral dataset in the terms of mean square error (MSE) and classification accuracy.

Comparative algorithms	MSE					Accuracy (%)	
	Best	Worst	Mean	Best		Worst	Mean
ABC-MLP	0.081348	0.099784	0.091211	90.566		77.358	84.371
BAT-MLP	0.069138	0.155741	0.099395	89.623		68.868	83.553
BBO-MLP	0.080143	0.096075	0.088869	88.679		84.906	86.730
FF-MLP	0.084314	0.099139	0.092277	87.736		84.906	86.384
FPA-MLP	0.092432	0.100673	0.096533	88.679		83.962	86.730
GA-MLP	0.080455	0.086119	0.083506	88.679		83.962	85.786
MBO-MLP	0.088745	0.119880	0.102397	90.566		76.415	85.660
PSO-MLP	0.099944	0.116638	0.108178	90.566		77.358	85.031
HOS-MLP	0.046793	0.078007	0.062629	93.670		91.139	92.405

**Table 6:** Comparison of accuracy (%) for the twelve classification algorithms. Bold values represent the best results.

Dataset	NB	BayesNet	SVM	MLP	KNN	AdaboostM1	Bagging	FLR	RF	FURIA	LMT	HOS-MLP
Breast cancer	94.95	96.21	95.79	95.37	95.79	94.53	94.95	89.49	95.79	94.95	96.21	96.833
Diabetes	77.01	78.16	80.84	74.32	72.79	77.01	77.77	67.04	76.24	77.39	79.31	77.840
Saheart	70.12	70.12	72.72	66.88	60.38	71.42	70.12	64.28	64.93	74.67	74.02	74.842
Vertebral	72.5	78.75	88.75	90.00	81.25	85.00	88.75	87.5	90.00	83.75	90.00	92.405

## Data Availability

No data were used to support this study.
